# Isolation of a New Natural Product and Cytotoxic and Antimicrobial Activities of Extracts from Fungi of Indonesian Marine Habitats

**DOI:** 10.3390/md9030294

**Published:** 2011-02-25

**Authors:** Kustiariyah Tarman, Ulrike Lindequist, Kristian Wende, Andrea Porzel, Norbert Arnold, Ludger A. Wessjohann

**Affiliations:** 1 Department of Pharmaceutical Biology, Ernst-Moritz-Arndt-University Greifswald, Friedrich-Ludwig-Jahn-Strasse 17, 17487 Greifswald, Germany; E-Mails: lindequi@uni-greifswald.de (U.L.); wende@uni-greifswald.de (K.W.); 2 Department of Aquatic Product Technology, Faculty of Fisheries and Marine Sciences, Bogor Agricultural University, Jl. Agathis 1, 16680 Darmaga-Bogor, Indonesia; 3 Department of Bioorganic Chemistry, Leibniz Institute of Plant Biochemistry, Weinberg 3, 06120 Halle (Saale), Germany; E-Mails: aporzel@ipb-halle.de (A.P.); narnold@ipb-halle.de (N.A.); wessjohann@ipb-halle.de (L.A.W.)

**Keywords:** Indonesian marine fungi, algicolous fungi, antifungal, bioactive compounds

## Abstract

In the search for bioactive compounds, 11 fungal strains were isolated from Indonesian marine habitats. Ethyl acetate extracts of their culture broth were tested for cytotoxic activity against a urinary bladder carcinoma cell line and for antifungal and antibacterial activities against fish and human pathogenic bacteria as well as against plant and human pathogenic fungi. The crude extract of a sterile algicolous fungus (KT31), isolated from the red seaweed *Kappaphycus alvarezii* (Doty) Doty ex P.C. Silva exhibited potent cytotoxic activity with an IC_50_ value of 1.5 μg/mL. Another fungal strain (KT29) displayed fungicidal properties against the plant pathogenic fungus *Cladosporium cucumerinum* Ell. et Arth. at 50 μg/spot. 2-Carboxy-8-methoxy-naphthalene-1-ol (**1**) could be isolated as a new natural product.

## 1. Introduction

Fungi produce a vast range of secondary metabolites [1]. Some of these are high-value products with pharmaceutical applications such as penicillins [2]. More specifically, marine fungi are also believed to be prolific resources of natural products [3–6]. However, their potential has not yet been fully investigated. Unlike the terrestrial fungi, which were initially exploited for drug discovery, marine fungi have attracted great attention as considerable resources only since the late 1980s [6]. Furthermore, it was reported that the corresponding chemistry of marine fungi was structurally diverse and related to that of terrestrial fungi [7].

Marine fungi comprise of an estimated 1500 species, excluding those that form lichens [8]. This number is low compared to the number of described and estimated terrestrial fungi (over 250,000) [9]. So far, less than 500 filamentous higher marine fungi have been described and only 79 are associated with algae as parasites or symbionts, and 18 with animal hosts [10]. A number of interesting compounds, such as cytoglobosins [11] and halovirs [12], had been isolated from marine fungi. Hence, we consider that there are numerous marine fungi containing further remarkable structures as well as bioactive compounds.

Although diverse, the distribution of marine fungi in the tropics has not been explored as thoroughly as in the temperate zone [13]. Nevertheless, inventory data for the marine fungi investigated in several tropical countries such as Thailand [14], Palau Islands [13], Singapore [15,16], Brunei [17], Malaysia [18–21] and Siargao Island, Philippines [22] are available. Other tropical regions have been explored even less, such as the Indonesian archipelago. This study, therefore, focuses on the investigation of marine fungi derived from different sources throughout Indonesian marine habitats. We report the isolation and structure elucidation of a new natural product (**1**) and the screening for bioactive compounds of fungal crude extracts.

## 2. Results and Discussion

### 2.1. Cultivation and Identification

The marine fungi investigated in this study were predominantly isolated from red seaweed (marine alga). Three strains were isolated from *Kappaphycus alvarezii* (Doty) Doty ex P.C. Silva (synonym: *Kappaphycus cottonii* (Weber-van Bosse) Doty ex P.C. Silva), two strains from *Eucheuma edule* (Kützing) Weber-van Bosse and one strain from *Gracilaria* sp. Surface sterilization of the algae followed by cultivation using agar media containing antibiotics was the best way to isolate pure strains of fungi.

All marine fungi were cultivated using different culture media to induce sporulation. Finally, some fungi producing spores could be identified by their morphological characteristics. Thus, three isolates were identified as *Aspergillus* sp. (KT13), *Lasiodiplodia theobromae* (Pat.) Griffon et Maubl. (KT26) and *Epicoccum nigrum* Link (KT28). Two algicolous isolates were identified as *Xylaria psidii* J.D. Rogers & Hemmes (KT30) and *Coniothyrium* sp. (KT33). Six strains could not be identified, five of which did not form spores under any of the applied culture conditions.

As it is difficult to obtain sexually reproducing forms, molecular biology-based methods such as sequencing rDNA can be a relevant strategy [6]. The rapid taxonomic assessment of fungal strains might be useful for drug discovery studies based on such organisms because it could reduce the risk of repetitive isolation of known substances. In the future this task will require more attention.

Salinity is one of the environmental factors that affect fungal growth as well as production of secondary metabolites. In an attempt to select the suitable culture conditions for the production of bioactive compounds, the culture medium salinity was varied using marine salt. Results from these tests were taken into account in further cultivation of each strain.

### 2.2. Biological Activity of Fungal Isolates

#### 2.2.1. Antibacterial Activity against Gram-Positive Bacteria

Crude extracts isolated from culture broth and mycelia were tested for their ability to inhibit growth of human and fish pathogenic bacteria. Most ethyl acetate extracts isolated from culture broth exhibited growth arresting activity against the test organisms. In sharp contrast to the ethyl acetate extracts, ethanol extracts isolated from culture broth as well as dichloromethane, methanol and water extracts from dried mycelia showed no activity. Dichloromethane extracts of KT30 and KT31 displayed an exception to this rule. Thus, further investigations were focused on the ethyl acetate extracts.

Table 1 presents the antibacterial activity of the ethyl acetate extracts of fungal culture broth against the Gram-positive bacteria *Bacillus subtilis* and *Staphylococcus aureus. Aspergillus* sp. (KT13) was the most active fungus against both bacteria with inhibition zone diameters in the range of 24 to 34 mm followed by strains KT31, KT03, KT19, *Lasiodiplodia theobromae* (KT26), and KT29, which were moderately active from both freshwater and seawater cultures.

However, salt concentration influenced the fungal cultures. As shown in Table 1, ethyl acetate extracts of isolates KT19, *L. theobromae* (KT26), and KT29 from seawater cultures showed a higher activity than those from the freshwater cultures and *vice versa* for the other strains. Salinity had a significant effect on the activity of ethyl acetate extracts of strain KT15. The extract isolated from seawater culture showed no activity against the test organisms in our test systems.

#### 2.2.2. Antibacterial Activity against Gram-Negative Bacteria

Agar diffusion assays of the ethyl acetate extracts showed that the growth of Gram-positive bacteria was more strongly inhibited than that of Gram-negative bacteria. Two strains, KT15 and *Coniothyrium* sp. (KT33), showed no activity against any of the test organisms. As presented in Table 2, extracts of strain KT19 from both culture conditions possessed antibacterial activity with inhibition zones in the range of 13–16 mm and 8–14 mm against *Escherichia coli* and *Pseudomonas aeruginosa*, respectively. However, extract of *X. psidii* (KT30) cultivated in freshwater medium was in fact the most active against *E. coli* and *P. aeruginosa* with inhibition zones of 23 and 13 mm, respectively. Some strains (KT03, KT19 and KT29) showed better activity when cultivated in seawater medium.

Remarkable activity was also exhibited by strain KT31. The ethyl acetate extract of isolate KT31 which was cultivated in seawater medium showed weak activity against *E. coli* with the inhibition zone of 10.6 mm, whereas the extract from freshwater culture was moderately active with the inhibition zone of 18.4 mm.

#### 2.2.3. Antibacterial Activity against Fish Pathogenic Bacteria

The ethyl acetate extracts of the culture broth as well as extracts of the mycelial biomass were tested against the Gram-negative, fish pathogenic bacteria *Vibrio anguillarum*, *Aeromonas salmonicida* and *Yersinia ruckeri.* These three bacteria are known as pathogens for marine and freshwater fish. The diseases caused by these microorganisms are commonly known as vibriosis, furunculosis and enteric red mouth disease, respectively.

Compared to antibacterial activity against *E. coli* and *P. aeruginosa*, the respective strains showed similar effects against the fish pathogenic bacteria (see Tables 2 and 3). However, the effects against fish pathogenic bacteria tended to be stronger, particularly against *V. anguillarum. Y. ruckeri* was the most resistant fish pathogenic test organism. As can be seen in Table 3, most of the ethyl acetate extracts isolated from the freshwater fungal culture inhibited the growth of fish pathogenic bacteria.

*X. psidii* (KT30) exhibited the highest activity observed against the fish pathogenic bacteria tested, followed by strain KT19. However, *X. psidii* (KT30) was more active when cultivated in freshwater medium, whereas fungal strain KT19 was most productive in salt water medium. Interestingly, in the above-mentioned conditions, these two strains showed similar effects against *Y. ruckeri*. Moreover, isolates of *Aspergillus* sp. (KT13), strains KT29 and KT31 exhibited similar activities.

Strains KT19 and KT29 displayed strong antibacterial activity when cultivated in seawater medium. There were no significant effects found for strains KT03 and KT32.

#### 2.2.4. Antifungal Activity against *Candida maltosa* and *Cladosporium cucumerinum*

Antifungal activity was observed less often among the extracts tested than antibacterial activity. From the eleven isolates only four strains exhibited antifungal properties against *Candida maltosa* (see Table 4).

Both ethyl acetate and methylene chloride (DCM) extracts of *X. psidii* (KT30) and strain KT31 showed antifungal activity, although DCM extracts were less active than the EtOAc extracts. From Table 4 it can be concluded that again *X. psidii* (KT30) was the most active organism against *Candida*. Strains KT19 and KT29, cultivated in seawater medium, showed similar activity as against human and fish pathogenic bacteria.

The ethyl acetate extracts of fungal culture broth were tested for their fungicidal properties against the phytopathogenic fungus *Cladosporium cucumerinum*. Most of the extracts showed fungicidal effects at 400 μg/78.5 mm^2^ spot. The inhibition of each active extract is listed in Table 5.

#### 2.2.5. Cytotoxic Activity

Table 6 presents the results of cytotoxicity assays against human bladder carcinoma cell line 5637 (ATCC HTB-9). *X. psidii* (KT30) and strain KT31 were strongly active in the cytotoxicity assays. Extracts of both strains exhibited IC_50_ values of 4 μg/mL and 1.5 μg/mL, respectively. Extracts obtained from seawater culture were less active. In the case of KT 30 and KT31 extracts, IC_50_ values were found to be 15 μg/mL and 14 μg/mL. However, these values represent in all cases a comparable high toxicity to many other fungal (and plant) crude extracts. Even though, it was not possible to identify the active principle until now. Further studies to reveal the compounds responsible shall be needed in order to identify the toxic principle in KT30 and KT31.

### 2.3. Isolation of a New Natural Product

From fungus KT29 one compound (**1**) was isolated and investigated further to determine its chemical structure. It was obtained as brown crystals by evaporation of methanol solution. The molecular formula of compound **1** was determined to be C_12_H_10_O_4_ by HR-FTICR-MS with the molecular ion peaks at *m/z* 241.04721 ([M + Na]^+^) in positive-ion mode and 217.05042 ([M − H]^−^) in negative-ion mode.

In order to elucidate the compound’s structure, NMR measurements were carried out in CD_3_OD. Interpretation of ^1^H and ^13^C-NMR spectra confirmed the presence of 12 carbon atoms and 10 proton signals, including methyl and hydroxyl groups. The presence of hydroxyl groups was confirmed by the IR spectrum showing an absorption band at 3439 cm^−1^. The IR (KBr) spectrum also exhibits the presence of a carbonyl group (1623 cm^−1^), aromatic system (1584 cm^−1^) and olefinic bond (1384 cm^−1^). The complete structural elucidation of **1** as well as the unambiguous assignment of all ^1^H and ^13^C NMR signals was based on 2D NMR experiments (HMBC, HSQC and ROESY). In particular, the attachment of the OH group at C-1 and not at C-2 is based on the HMBC correlation of H-3 with the carbonyl signal at 176.9 ppm (C-11) as well as on the high-field shift of C-9 (117.8 ppm) due to two OR substituents in *ortho* position. The ^1^H and ^13^C-NMR data of compound **1** are presented in Table 7.

**Figure 1.**
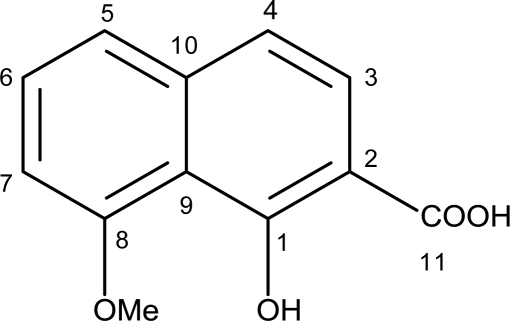
Molecular structure of compound **1 (**C_12_H_10_O_4_).

According to the spectroscopic data, compound **1** was determined as 2-carboxy-8-methoxynaphthalene-1-ol (Figure 1). To date, this compound was only known as an intermediate in the synthesis of a naphthalene carboxylic acid (C_20_H_18_O_8_) using naphthalenediol (C_10_H_8_O_2_) as starting material [23,24]. Thus, the discovery of compound **1** as a natural product is new.

In agar diffusion assays, compound **1** showed no antimicrobial activity against *Staphylococcus aureus*, *Pseudomonas aeruginosa*, *Escherichia coli* at 100 μg/disc and *Candida maltosa* at 200 μg/disc. Compound **1** was also tested *in vitro* against the human bladder carcinoma cell line 5637 with etoposide as the positive control. The results showed that the compound possesses a negligible cytotoxic activity with an IC_50_ value of 0.34 mM, compared to etoposide with an IC_50_ value of 0.6 μM. Against *Cladosporium cucumerinum*, the compound was not active at a concentration of 200 μg/spot. In contrast to our results, many other naphthalene derivatives, mainly naphthoquinones, possess strong antibacterial, antiviral and further activities [25]. This discrepancy might be due to the following reasons: Compound **1** does not possess a chinoid structure (as do many of the active naphthalene derivatives), and it can be easily metabolized by esterification or etherification of the hydroxyl group at C-1 and the carboxyl group at C-2. The carboxyl group itself renders the compound quite polar and thereby massively reduces transmembrane transport, resulting in a low (cellular) availability.

In addition to the described new substance another isolated compound displayed stronger activity. Unfortunately due to a very low yield the structural elucidation of this compound has not been completed.

## 3. Experimental Section

### 3.1. General

NMR spectra were recorded on a Varian VNMRS at 600 MHz for ^1^H and at 150 MHz for ^13^C. The samples were dissolved in CD_3_OD. Chemical shifts were referenced to internal TMS (δ = 0 ppm, ^1^H) and CD_3_OD (δ = 49.0 ppm, ^13^C), respectively. ESI-MS and HR-ESI-MS were performed using Bruker Apex III 70 e FT-ICR-MS; spectra were expressed in *m/z*. Analytical TLC was performed on Merck precoated silica gel 60 F_254_. Spots were visualized by UV light (λ 254 and 366 nm) and spraying with anisaldehyde/sulfuric acid reagent (1% in a solution of 10 mL of AcOH in 10 mL of a 15% methanolic H_2_SO_4_) followed by heating. Column chromatography was carried out using Sephadex LH-20 (Amersham Biosciences) using methanol as mobile phase. Fractions were collected and processed responding to their chemical composition. IR Spectra were measured on a Perkin Elmer System 2000 FT-IR; wave numbers were expressed in cm^−1^.

### 3.2. Fungal Isolates

Fungal host samples were collected from Indonesian marine and coastal areas, located in East and West Java, and North Jakarta in April-May 2007 and from South Sulawesi in May 2008. The samples included driftwoods, mollusc shells, sand foam and algae. Eleven fungal strains were isolated consisting of six algicolous fungi (voucher No. KT28, KT29, KT30, KT31, KT32, KT33), two lignicolous fungi (voucher No. KT03 and KT26), two isolates from sandy habitats (voucher No. KT13 and KT19) and one strain from mollusc shell (voucher No. 15). The isolates are deposited in the Institute of Pharmacy, Greifswald University, Germany. Five isolates were morphologically identified as *Aspergillus* sp. (KT13), *Lasiodiplodia theobromae* (Pat.) Griffon et Maubl. (KT26), *Epicoccum nigrum* Link (KT28), *Xylaria psidii* J.D. Rogers & Hemmes (KT30), *Coniothyrium* sp. (KT33). Six other strains have not been identified until now.

### 3.3. Fermentation, Extraction and Isolation

Cultivation was carried out in liquid shake cultures in 500 mL Erlenmeyer flasks containing 200 mL of *Hagem* medium (0.5 g of ammonium succinate; 0.5 g of KH_2_PO_4_; 0.5 g of MgSO_4_.7H_2_O; 0.5 mL of FeCl_3_ (1%); 5 g of glucose; 5 g of malt extract; 1000 mL of aqua destillata; pH 7.5). The sterilized media were inoculated with a homogenized pre-culture of fungal strain and incubated on a rotary shaker (120 rpm) for 14–21 days at room temperature.

Finally, mycelia and culture broth were separated, and the filtered culture broth was extracted with EtOAc (3 × 500 mL). The resulting EtOAc broth extracts were dried in a vacuum evaporator and the residue was extracted with ethanol. The mycelia were lyophilized and subsequently extracted with DCM, MeOH and water using a Soxhlet apparatus. After drying (Na_2_SO_4_), the organic layers were concentrated using a vacuum evaporator to afford the respective crude extracts.

All fungal extracts were screened for their biological activities. However, only the ethyl acetate extracts of the fungal culture broth exhibited considerable antibacterial and cytotoxic activities. Therefore, the present study was focused on EtOAc broth extracts.

The ethyl acetate extract (525 mg) of KT29 was fractionated on Sephadex LH-20 and using 100% MeOH. Based on TLC analysis, subfractions were combined into 10 fractions. On TLC, fraction F5 showed a major band with a blue fluorescence both in short and long wavelength UV detection. Subsequently, F5 was refractionated on Sephadex LH-20 using methanol as mobile phase to afford compound **1** (13.9 mg; R_f_ = 0.45 on Si60 F_254_ plate (Merck) using toluene/ethyl formate/formic acid, 10:5:3, v/v). The purity of the compound was confirmed by RP-HPLC (YMC ODS-A 120, 5 μm) with a linear gradient of 2–100% aqueous ACN (0.1% TFA) for 35 min; the retention time was 13.833 min.

### 3.4. Bioassays

Agar diffusion assays to determine antibacterial activity were performed according to the Kirby-Bauer method as described by Boyle *et al.* [26]. Ampicillin, gentamicin and oxytetracycline were used as positive controls for antibacterial tests and nystatin and benomyl for antifungal tests. Gram-positive bacteria *Bacillus subtilis* ATCC 6051 and *Staphylococcus aureus* ATCC 6538 as well as Gram-negative bacteria *Escherichia coli* ATCC 11229, *Pseudomonas aeruginosa* ATCC 22853, *Vibrio anguillarum* DSMZ 11323, *Aeromonas salmonicida* ATCC 51413 and *Yersinia ruckeri* ATCC 29493 were used as test organisms. *Candida maltosa* SBUG 700 and *Cladosporium cucumerinum* were used as test organisms in antifungal assays.

The activity against the phytopathogenic fungus *C. cucumerinum* was carried out by the method of Gottstein *et al.*, a semiquantitative test that allows a relative estimation of the activity of the compounds with similar diffusion characteristics [27]. Briefly, the crude extracts were loaded on TLC plates (glass plate, 20 cm × 20 cm, silica gel 60 HF_254_, thickness 0.5 mm) using a microliter syringe. Loaded samples give 10 mm diameter (area of 78.5 mm^2^). The dried plates were sprayed with 10 mL spore suspension of *C. cucumerinum* (spore density *ca.* 2.5 × 10^6^ spores/mL) and dried at room temperature for about 15 min. The dried plates were then placed in a TLC chamber containing moist filter paper and the fungus was cultivated in an incubator at 25 °C for two days. Fungicidal activity was measured as inhibition area (mm^2^). A larger area correlates with higher activity and mobility of fungicidal constituents.

Cytotoxic activity of the crude extracts was determined by Neutral Red Uptake Assay (NRU-Test) according to Bohrenfreund and Puerner [28] using the cultivated human bladder carcinoma cell line 5637 (ATCC HTB-9).

For assays, 5637 cells were seeded at 2.5 × 10^3^ cells/mL in 96-well plates (TPP; Trasadingen, CH) and left undisturbed overnight. After washing, fresh medium containing test substances or controls (etoposide, vehicle) was added. Extracts and controls were diluted in assay medium using a stock solution (20 mg/mL in vehicle). Final vehicle concentration did not exceed 0.05%. Cells were allowed to grow for 72 h at 37 °C. Finally, plates were washed and incubated with RPMI 1640 containing 3.3 μg/mL neutral red for 3 h. After removing the supernatant and extensive washing, cells were lysed in acidic ethanol and OD at 540 nm was measured. Cell viability was calculated as percentage of vehicle control after background reduction. All experiments were carried out twice with six replicates for each concentration tested. Where applicable, IC_50_ values were calculated by linear regression using MS Excel.

## 4. Conclusions

Of the eleven marine-derived fungi, five were found to be a potent source of bioactive natural products. The ethyl acetate extracts of the fungal culture broth of *Xylaria psidii* (KT30) and *Mycelium sterilium* (KT31) exhibited considerable antibacterial and cytotoxic activities, but only minor antifungal activity. In some marine fungal strains freshwater-based medium can be used to enhance the production of bioactive secondary metabolites. Depending on the strain, there were differences in the strength of the bioactivity between freshwater and seawater cultures. A 2-carboxy-8-methoxy-naphthalene-1-ol was isolated as a new natural compound but was found to be inactive in antibacterial test probably due to its high polarity. Further research shall be needed to identify the active principle within the extracts from *X. psidii* (KT30) and strain KT31.

## Figures and Tables

**Table 1.** Antibacterial activity of the ethyl acetate extracts against Gram-positive bacteria, measured as inhibition zones (mm) in the agar diffusion assay.

**Fungal strain**	***Bacillus subtilis***		***Staphylococcus aureus***	
	**Freshwater culture**	**Seawater culture**	**Freshwater culture**	**Seawater culture**
*Mycelium sterilium* (KT03)	10.5 ± 1.7	12.4 ± 0.5	26.3 ± 1.0	17.5 ± 0.6
*Aspergillus* sp. (KT13)	31.5 ± 2.4	25.1 ± 0.9	31.6 ± 0.5	24.8 ± 1.0
Unidentified strain (KT15)	23.0 ± 1.4	-	18.3 ± 1.0	-
*Mycelium sterilium* (KT19)	9.4 ± 0.5	22.9 ± 1.6	10.4 ± 0.8	21.0 ± 0.8
*Lasiodiplodia theobromae* (KT26)	12.8 ± 0.7	20.0 ± 1.8	11.9 ± 1.3	18.0 ± 0.0
*Epicoccum nigrum* (KT28)	-	8.4 ± 0.5	8.8 ± 0.5	7.5 ± 0.6
*Mycelium sterilium* (KT29)	9.8 ± 1.0	20.8 ± 1.0	9.8 ± 1.5	13.9 ± 0.3
*Xylaria psidii* (KT30)	12.9 ± 0.3	14.4 ± 1.0	14.9 ± 0.3	7.5 ± 0.6
*Mycelium sterilium* (KT31)	22.9 ± 0.6	9.9 ± 0.9	20.5 ± 1.3	10.8 ± 0.5
*Mycelium sterilium* (KT32)	13.0 ± 0.4	8.3 ± 0.5	12.0 ± 0.0	7.3 ± 0.5
*Coniothyrium* sp. (KT33)	8.3 ± 1.0	8.5 ± 0.5	11.6 ± 0.5	9.3 ± 1.7
Ampicillin (10 μg/disc)	7.5 ± 0.7	7.5 ± 0.7		
Ampicillin (50 μg/disc)			32.5 ± 0.7	32.5 ± 0.7

Extracts tested 2 mg/6 mm disc; Inhibition zone in mm including disc, expressed as Mean ± SD (*n* = 4);

-: No inhibition zone measured.

**Table 2.** Antibacterial activity of ethyl acetate extracts against Gram-negative bacteria, measured as inhibition zones (mm) in the agar diffusion assay.

**Fungal strain**	***Escherichia coli***		***Pseudomonas aeruginosa***	
	**Freshwater culture**	**Seawater culture**	**Freshwater culture**	**Seawater culture**
*Mycelium sterilium* (KT03)	8.6 ± 0.5	10.4 ± 0.5	7.9 ± 0.3	11.9 ± 0.3
*Aspergillus* sp. (KT13)	9.4 ± 0.5	-	-	-
Unidentified strain (KT15)	-	-	-	-
*Mycelium sterilium* KT19	13.9 ± 0.9	14.1 ± 1.3	8.0 ± 0.0	13.9 ± 0.3
*Lasiodiplodia theobromae* (KT26)	81 ± 0.3	-	13.4 ± 0.8	8.1 ± 0.6
*Epicoccum nigrum* (KT28)	7.8 ± 1.0	-	-	-
*Mycelium sterilium* (KT29)	7.6 ± 0.5	12.5 ± 1.3	6.6 ± 0.5	12.6 ± 0.5
*Xylaria psidii* (KT30)	22.4 ± 0.8	8.9 ± 0.3	12.9 ± 0.3	-
*Mycelium sterilium* (KT31)	18.4 ± 0.5	10.6 ± 0.5	-	-
*Mycelium sterilium* (KT32)	8.9 ± 0.3	8.3 ± 0.5	-	-
*Coniothyrium* sp. (KT33)	-	7.0 ± 0.0	-	-
Ampicillin (50 μg/disc)	17.8 ± 0.4	17.8 ± 0.4		
Gentamicin (10 μg/disc)			10.3 ± 0.5	10.3 ± 0.5

Extracts tested 2 mg/6 mm disc; Inhibition zone in mm including disc, expressed as Mean ± SD (*n* = 4);

-: No inhibition zone measured.

**Table 3.** Antibacterial activity of ethyl acetate extracts against fish pathogenic bacteria, measured diffusion assay.

**Fungal strain**	***Vibrio anguillarum***		***Aeromonas salmonicida***		***Yersinia ruckeri***	
	**Freshwater culture**	**Seawater culture**	**Freshwater culture**	**Seawater culture**	**Freshwater culture**	**Seawater culture**
*Mycelium sterilium* (KT03)	13.3 ± 0.9	12.4 ± 0.5	10.4 ± 0.8	12.0 ± 1.4	-	-
*Aspergillus* sp. (KT13)	21.6 ± 0.8	17.8 ± 0.5	19.3 ± 1.0	16.9 ± 0.6	-	-
Unidentified strain (KT15)	16.3 ± 1.3	-	11.4 ± 1.3	-	-	-
*Mycelium sterilium* (KT19)	17.5 ± 0.6	18.3 ± 1.0	8.9 ± 0.3	16.0 ± 0.8	-	24.8 ± 1.0
*Lasiodiplodia theobromae* (KT26)	17.1 ± 1.0	10.9 ± 0.3	13.4 ± 0.5	7.3 ± 0.5	8.5 ± 0.6	-
*Epicoccum nigrum* (KT28)	15.0 ± 0.8	12.6 ± 0.8	8.0 ± 0.8	9.1 ± 0.6	8.3 ± 0.5	-
*Mycelium sterilium* (KT29)	17.0 ± 0.8	21.3 ± 1.0	12.8 ± 1.0	18.4 ± 2.3	7.0 ± 0.0	-
*Xylaria psidii* (KT30)	21.3 ± 1.0	16.3 ± 0.5	22.4 ± 1.7	9.3 ± 0.5	25.1 ± 0.6	8.1 ± 0.9
*Mycelium sterilium* (KT31)	18.8 ± 0.5	21.3 ± 1.0	19.3 ± 1.0	13.8 ± 0.3	10.6 ± 0.5	-
*Mycelium sterilium* (KT32)	14.0 ± 0.4	14.8 ± 0.6	13.8 ± 0.5	13.3 ± 1.0	-	-
*Coniothyrium* sp. (KT33)	18.9 ± 1.5	17.4 ± 0.5	10.8 ± 0.5	-	-	-
Oxytetracycline (30 μg/disc)	20.9 ± 1.0	20.9 ± 1.0	24.9 ± 0.6	24.9 ± 0.6	12.6 ± 0.5	12.6 ± 0.5

Extracts tested 2 mg/6 mm disc; Inhibition zone in mm including disc, expressed as Mean ± SD (*n* = 4);

-: No inhibition zone measured.

**Table 4.** Antibacterial activity of EtOAc and DCM crude extracts against *Candida maltosa*, measured as inhibition zones (mm) in the agar diffusion assay.

**Fungal strain**	***Candida maltosa***	
	**Freshwater culture**	**Seawater culture**
*Mycelium sterilium* (KT03)	-	-
*Aspergillus* sp. (KT13)	-	-
Unidentified strain (KT15)	-	-
*Mycelium sterilium* (KT19)	-	13.5 ± 1.3
*Lasiodiplodia theobromae* (KT26)	-	-
*Epicoccum nigrum* (KT28)	-	-
*Mycelium sterilium* (KT29)	-	13.6 ± 0.5
*Xylaria psidii* (KT30)	22.3 ± 0.5	-
*Xylaria psidii* (KT30) *	15.6 ± 0.5	-
*Mycelium sterilium* (KT31)	13.5 ± 1.7	-
*Mycelium sterilium* (KT31) *	11.0 ± 0.0	-
*Mycelium sterilium* (KT32)	-	-
*Coniothyrium* sp. (KT33)	-	-
Nystatin (50 μg/disc)	23.4 ± 1.1	23.4 ± 1.1

Extracts tested 2 mg/6 mm disc; Inhibition zone in mm including disc, expressed as Mean ± SD (*n* = 4);

-: No inhibition zone measured;

* Dichloromethane (DCM) extracts.

**Table 5.** Inhibition area in mm^2^ of EtOAc extracts after application of 50 μg to 400 μg. A larger area correlates with higher activity and mobility of fungicidal constituents.

**Fungal strain**	**Concentration (μg/spot)**			
	**50**	**100**	**200**	**400**
*Mycelium sterilium* (KT03)	-	-	-	-
*Mycelium sterilium* (KT03) ^#^	-	-	-	-
*Aspergillus* sp. (KT13)	-	-	-	-
*Aspergillus* sp. (KT13) ^#^	-	-	-	78.5
Unidentified strain KT15	-	-	-	28.3
Unidentified strain (KT15) ^#^	-	-	-	-
*Mycelium sterilium* (KT19) ^#^	-	-	-	50.2
*Lasiodiplodia theobromae* (KT26)	-	-	-	-
*Lasiodiplodia theobromae* (KT26) ^#^	-	-	-	-
*Mycelium sterilium* (KT29) ^#^	56.7	78.5	78.5	95.0
*Xylaria psidii* (KT30)	-	78.5 ^‡^	19.6	78.5
*Mycelium sterilium* (KT31)	-	-	28.3	95.0
*Mycelium sterilium* (KT32)	-	-	78.5 ^‡^	78.5
*Coniothyrium* sp. (KT33)	-	-	78.5 ^‡^	78.5
Benomyl 80 ng/spot	78.5			

# Cultivated in seawater medium;

-: No inhibition zone measured;

‡ Fungistatic activity.

**Table 6.** Cytotoxic activity of EtOAc extracts against human bladder carcinoma cell line 5637.

**Strain**	**IC_50_ (μg/mL)**	
	**Freshwater culture**	**Seawater culture**
*Mycelium sterilium* (KT03)	-	-
*Aspergillus* sp. (KT13)	50	-
Unidentified strain (KT15)	-	-
*Mycelium sterilium* (KT19)	-	50
*Lasiodiplodia theobromae* (KT26)	-	-
*Epicoccum nigrum* (KT28)	48	56
*Mycelium sterilium* (KT29)	-	-
*Xylaria psidii* (KT30)	4	15
*Mycelium sterilium* (KT31)	1.5	14
*Mycelium sterilium* (KT32)	18	50
*Coniothyrium* sp. (KT33)	-	60
Etoposide	0.35	0.35

-: No activity observed.

**Table 7.** ^1^H- and ^13^C-NMR data of compound **1** in CD_3_OD (recorded at 600/150 MHz in CD_3_OD; δ in ppm, *J* in Hz).

**Position**	**δ ^13^C (ppm)**	**δ ^1^H (ppm)**	**HMBC (H to C#)**	**selected ROESY**
1	162.4	-		
2	113.7	-		
3	128.9	7.89 d (8.5)	1, 10, 11	
4	117.9	7.13 d (8.5)	2, 5, 9	H-5
5	121.5	7.29 d (8.2)	4, 7, 9	H-4
6	129.0	7.37 dd (8.2/7.8)	8, 10	
7	106.4	6.86 d (7.8)	5, 9	8-OMe
8	159.8	-		
9	117.8	-		
10	140.9	-		
11	176.9	-		
OMe	56.3	3.93	8	H-7
